# The time has come for big changes to improve research funding

**DOI:** 10.1371/journal.pbio.3003754

**Published:** 2026-04-06

**Authors:** Peter Kolarz

**Affiliations:** Research on Research Institute, Department of Science, Technology, Engineering and Public Policy, University College London, London, United Kingdom

## Abstract

The competitive research funding system is at breaking point. This Perspective argues that innovations to address ongoing problems are needed on a grander scale than ever before, but this will not suffice. What we need is whole system transformation.

## Teaser

The competitive research funding system is at a breaking point. Innovations to address ongoing problems are needed on a grander scale than ever before, but even this will not suffice to fix a struggling system. What we need is a whole system transformation.

The international research funding and proposal-assessment machine is creaking. A combination of old and new problems (including old ones that are intensifying) means we need to implement solutions faster and on grander scales. But this alone will not do: a wider system transformation is also needed.

The competitive research funding system faces many challenges. Most critically, the peer review burden is increasing [1,2]. Besides the strain on researchers’ productivity, this leads to several knock-on problems, including funders struggling to find enough available reviewers [3]. Peer review is also an imperfect arbiter of quality: the commonly practiced process of external peer review followed by panel review does well at identifying sub-standard work, as well as the very highest peaks of quality, but it tends to produce arbitrary outcomes in the upper midfield of the quality spectrum [4–6]. In addition, peer review can put particularly novel or interdisciplinary perspectives at a disadvantage. Radical, ‘breakthrough’ ideas tend to be less able to garner positive consensus from multiple reviewers, so they lose out to more incremental, ‘safe’ research proposals [7,8]. Beyond novelty, it is also unclear how well peer review is suited to identifying and rewarding potential societal use and relevance of proposed projects [9].

The rise of artificial intelligence (AI) also poses a whole range of potential challenges. Writing an application is now less time-consuming, as parts of it can be automated (more so with the most recent rise of agentic AI tools), making it easier to apply for grants. However, the additional pressure that this places on the system is not just a burden-intensifier; it may also incentivize unethical use of AI throughout the process, from application writing to reviewing applications, writing reviews, and ranking/decision-making itself. The result could be a profound legitimacy crisis of the entire funding system.

There are, in short, ample reasons to move forward with significant reforms to the international competitive research funding system. Much is already being done: funders have trialed and implemented modifications to standard selection and decision-making processes, 38 of which are detailed in a 2023 report [10]. Some of these are simple tweaks that are already practiced widely, such as short pre-proposals, shortening application sections, or briefing panelists on how to apply criteria. But some even more radical interventions have now been practiced and trialed at length. Since the above-mentioned 2023 report, evidence of efficacy has mounted further, so it is time to move beyond experimentation and towards wider rollout.

Two examples of such interventions are distributed peer review (DPR) and partial randomization. DPR involves applicants to a scheme also acting as reviewers on that same scheme. In other words, applicants review each other. Evidence suggests gaming is remarkably rare and can be further mitigated by splitting applicants into two groups, where ‘pot A’ applicants only review those in ‘pot B’, and vice versa. Especially in large calls or those with a relatively limited thematic remit, the applicants themselves typically provide a suitably broad base of expertise. Aside from the obvious benefit of not having to recruit external reviewers, current evidence from various trials suggests DPR facilitates faster time-to-grant and more timely submission of reviews. DPR is also fully scalable and can improve reviewer diversity (especially by providing a larger number of reviews per application). Additionally, it provides a degree of demand management, as it discourages serial speculative resubmission of already-written applications [11,12].

The second approach, partial randomization (sometimes simply known as ‘lottery’), involves selecting proposals at random from a subset of submitted applications that have already passed a minimum quality threshold. It is an increasingly common way to reduce the effect of bias, enable the funding of riskier, more novel ideas, and reduce burden in the decision-making process. While some funders use it across larger selections of applications, it is commonly used as a tie-breaker or for a small set of applications that have already been judged to be of fundable quality, but where the budget is insufficient to fund all of them. Although it is usually a modest process change, evidence of its benefit is now substantial and further rollout is advisable [13].

Other interventions have not been as rigorously tested yet, so more experimentation is needed with a view to rapid rollout if positive findings emerge. For instance, a recent study suggested that randomization before review may provide substantial relief to the current system overload. Programs with exceptionally high demand might introduce a sign-up system, from which invitations to apply are then selected at random, ensuring application volumes do not overload the system. This approach may be worth introducing more widely alongside other more established demand management mechanisms (e.g., prohibiting multiple applications per person or within a certain timeframe). The recent study suggests that ‘lottery before review’ may also entail increased participation from women and reduced economic cost [14].

Many of these interventions will bring relief to an overburdened system and address many of the other challenges around peer review. However, they are unlikely on their own to solve them completely. This is because all these innovations would still exist in a hyper-competitive grant funding environment, where researchers depend on grant income for their job security in a research landscape that looks increasingly precarious in most places. The drivers for increased applications (and declining success rates, in turn leading to increased re-application, thus fueling the peer review overload) will likely be further exacerbated through the rise of agentic AI tools.

In addition to a wider rollout of verifiably beneficial innovations in grant funding, it is therefore critical to initiate wider system reforms: specifically, there is now a clear and present need to take the heat out of the intense competition for grants via appropriate incentive setting. Most obviously, where grant income or grant application rates are used as part of individual researchers' performance management, an overload of the peer review system is inevitable. In line with the wider movement for responsible research assessment, it is critical for higher-level decision makers to identify where such perverse incentives exist and to remove them.

More broadly, we need to consider strengthening institutions, teams, and groups as the main formative unit of how science is funded, rather than placing ‘superstar’ academics and their grant-winning skills at the heart of the academic enterprise (as well as forcing early career researchers to prioritize grant-winning over any other part of their emerging skillset). Shifting the balance from competitive project funding towards institutional funding is one possible approach here. This may also entail a move towards mission-based institutes (either standalone or within existing universities) where individual researchers’ funds to undertake research are somewhat implicit in their position and the institute's goals, while the institute's available budget is a function of collective endeavor and success. Critical in any such landscape shifts will be the work of initiatives such as DORA and CoARA, who push for responsible assessment practices and healthy incentive structures.

There will likely always be a role for grant funding. Where such direct competition of ideas makes sense, process experimentation and rollout of innovative funding approaches are the way forward. But the recent exacerbation of challenges around peer review means that person-centered, application-based competitive funding can no longer be the central driver and shaper of how the research world works. The time for systemic change is here.

## Abbreviations

AI: artificial intelligence
DPR: distributed peer review.
